# Chidamide in relapsed or refractory peripheral T cell lymphoma: a multicenter real-world study in China

**DOI:** 10.1186/s13045-017-0439-6

**Published:** 2017-03-15

**Authors:** Yuankai Shi, Bo Jia, Wei Xu, Wenyu Li, Ting Liu, Peng Liu, Weili Zhao, Huilai Zhang, Xiuhua Sun, Haiyan Yang, Xi Zhang, Jie Jin, Zhengming Jin, Zhiming Li, Lugui Qiu, Mei Dong, Xiaobing Huang, Yi Luo, Xiaodong Wang, Xin Wang, Jianqiu Wu, Jingyan Xu, Pingyong Yi, Jianfeng Zhou, Hongming He, Lin Liu, Jianzhen Shen, Xiaoqiong Tang, Jinghua Wang, Jianmin Yang, Qingshu Zeng, Zhihui Zhang, Zhen Cai, Xiequn Chen, Kaiyang Ding, Ming Hou, Huiqiang Huang, Xiaoling Li, Rong Liang, Qifa Liu, Yuqin Song, Hang Su, Yuhuan Gao, Lihong Liu, Jianmin Luo, Liping Su, Zimin Sun, Huo Tan, Huaqing Wang, Jingwen Wang, Shuye Wang, Hongyu Zhang, Xiaohong Zhang, Daobin Zhou, Ou Bai, Gang Wu, Liling Zhang, Yizhuo Zhang

**Affiliations:** 1 Department of Medical Oncology, Beijing Key Laboratory of Clinical Study on Anticancer Molecular Targeted Drugs, National Cancer Center/Cancer Hospital, Chinese Academy of Medical Sciences and Peking Union Medical College, Beijing, 100021 China; 2 Peking University Cancer Hospital and Institute, Beijing, China; 3 Jiangsu Province Hospital, Nanjing, China; 4 Guangdong General Hospital, Guangzhou, China; 5 West China Hospital, Sichuan University, Chengdu, China; 6 Zhongshan Hospital, Shanghai, China; 7 Shanghai Ruijin Hospital, Shanghai, China; 8 Tianjin Medical University Cancer Institute and Hospital, Tianjin, China; 9 The Second Hospital of Dalian Medical University, Dalian, China; 10 Zhejiang Cancer Hospital, Hangzhou, China; 11 Xinqiao Hospital, Third Military Medical University, Chongqing, China; 12 The First Affiliated Hospital, Zhejiang University, Hangzhou, China; 13 The First Affiliated Hospital of Soochow University, Suzhou, China; 14 Sun Yat-Sen University Cancer Center, Guangzhou, China; 15 Hematology Institute and Hospital, Chinese Academy of Medical Sciences and Peking Union Medical College, Tianjin, China; 16 Sichuan Provincial People’s Hospital, Chengdu, China; 17 Hunan Cancer Hospital, Changsha, China; 18 Shandong Provincial Hospital, Jinan, China; 19 Jiangsu Cancer Hospital, Nanjing, China; 20 Nanjing Drum Tower Hospital, Nanjing, China; 21 Tongji Hospital, Wuhan, China; 22 Fujian Provincial Cancer Hospital, Fuzhou, China; 23 The First Affiliated Hospital of Chongqing Medical University, Chongqing, China; 24 Union Hospital, Fujian Medical University, Fuzhou, China; 25 General Hospital of Nanjing Military Region, Nanjing, China; 26 Changhai Hospital, Shanghai, China; 27 The First Affiliated Hospital of Anhui Medical University, Hefei, China; 28 Sichuan Cancer Hospital and Institute, Chengdu, China; 29 Xijing Hospital, The Fourth Military Medical University, Xi’an, China; 30 Anhui Provincial Hospital, Hefei, China; 31 QiLu Hospital of Shandong University, Jinan, China; 32 Liaoning Cancer Hospital and Institute, Dalian, China; 33 Nanfang Hospital, Southern Medical University, Guangzhou, China; 34 The 307th Hospital of Chinese People’s Liberation Army, Beijing, China; 35 Fourth Hospital of Hebei Medical University (Tumor Hospital of Hebei Province), Shijiazhuang, China; 36 The Second Hospital of Hebei Medical University, Shijiazhuang, China; 37 Shanxi Provincial Cancer Hospital, Taiyuan, China; 38 The First Affiliated Hospital of Guangzhou Medical University, Guangzhou, China; 39 Tianjin People’s Hospital, Tianjin, China; 40 Beijing Tongren Hospital, Beijing, China; 41 The First Affiliated Hospital of Harbin Medical University, Harbin, China; 42 Peking University Shenzhen Hospital, Shenzhen, China; 43 The Second Affiliated Hospital Zhejiang University School of Medicine, Hangzhou, China; 44 Peking Union Medical College Hospital, Beijing, China; 45 The First Hospital of Jilin University, Changchun, China; 46 Wuhan Union Hospital of China, Wuhan, China

**Keywords:** Chidamide, Peripheral T cell lymphoma, Treatment, Chemotherapy

## Abstract

The efficacy and safety of chidamide, a new subtype-selective histone deacetylase (HDAC) inhibitor, have been demonstrated in a pivotal phase II clinical trial, and chidamide has been approved by the China Food and Drug Administration (CFDA) as a treatment for relapsed or refractory peripheral T cell lymphoma (PTCL). This study sought to further evaluate the real-world utilization of chidamide in 383 relapsed or refractory PTCL patients from April 2015 to February 2016 in mainland China. For patients receiving chidamide monotherapy (*n* = 256), the overall response rate (ORR) and disease control rate (DCR) were 39.06 and 64.45%, respectively. The ORR and DCR were 51.18 and 74.02%, respectively, for patients receiving chidamide combined with chemotherapy (*n* = 127). For patients receiving chidamide monotherapy and chidamide combined with chemotherapy, the median progression-free survival (PFS) was 129 (95% CI 82 to 194) days for the monotherapy group and 152 (95% CI 93 to 201) days for the combined therapy group (*P* = 0.3266). Most adverse events (AEs) were of grade 1 to 2. AEs of grade 3 or higher that occurred in ≥5% of patients receiving chidamide monotherapy included thrombocytopenia (10.2%) and neutropenia (6.2%). For patients receiving chidamide combined with chemotherapy, grade 3 to 4 AEs that occurred in ≥5% of patients included thrombocytopenia (18.1%), neutropenia (12.6%), anemia (7.1%), and fatigue (5.5%). This large real-world study demonstrates that chidamide has a favorable efficacy and an acceptable safety profile for refractory and relapsed PTCL patients. Chidamide combined with chemotherapy may be a new treatment choice for refractory and relapsed PTCL patients but requires further investigation.

**Electronic supplementary material:**

The online version of this article (doi:10.1186/s13045-017-0439-6) contains supplementary material, which is available to authorized users.

## Letter to the editor

Peripheral T cell lymphomas (PTCLs) are a set of rare and highly heterogeneous tumors derived from mature T cells or natural killer cells and are typically characterized by poor prognosis and aggressive clinical behavior [1]. PTCL accounts for 23 to 26% of all non-Hodgkin’s lymphoma (NHL) in China, which is significantly higher than the rates in Western countries [2, 3]. A consensus has not been reached on standard treatments for PTCL patients, and most commonly used traditional chemotherapy regimens are associated with a poor response [1, 4]. Moreover, a majority of patients may experience disease relapse even if they receive high-dose chemotherapy and autologous stem cell transplantation (ASCT) [5, 6]

Since 2009, the US Food and Drug Administration (FDA) has approved four new drugs for the treatment of relapsed or refractory PTCL, including the histone deacetylase (HDAC) inhibitors romidepsin and belinostat, the dihydrofolate reductase inhibitor pralatrexate, and the CD30 antibody-drug conjugate brentuximab vedotin for CD30-positive anaplastic large cell lymphoma (ALCL) patients [7, 8].

Chidamide, an innovative new drug independently developed in China, is designed to selectively inhibit the activity of HDAC1, 2, 3, and 10 following oral administration and was approved in December 2014 by the China Food and Drug Administration (CFDA) for the treatment of relapsed or refractory PTCL [9].

The efficacy and safety of chidamide have been demonstrated in a pivotal phase II clinical trial [10], yet further evaluation of its real-world utility is urgently needed. Therefore, we conducted a real-world multicenter efficacy and safety monitoring study to further test the clinical practice value of chidamide in relapsed or refractory PTCL patients in mainland China.

We analyzed 383 patients from April 2015 to February 2016. The cutoff date was February 19, 2016. The methods are shown in Additional file 1. The baseline characteristics of all patients are presented in Additional file 2.

For patients receiving chidamide monotherapy (*n* = 256), the overall response rate (ORR) and disease control rate (DCR) were 39.06 and 64.45%, respectively. In previous phase II study, the AITL patients received chidamide have a higher ORR of 50%. Higher ORR and superior survival were also observed for AITL patients received romidepsin and belinostat. In this real world study, AITL patients also tend to have higher ORR and DCR of 49.23% and 75.38% which were comparable with previous results. It has been reported that epigenetic regulation plays an important role in AITL pathogenesis, which may be relevant to more clinical benefits by HDAC inhibitors to AITL. The ORR and DCR seem higher for ALK+ ALCL patients receiving chidamide of 66.67% and 83.33%, but only 13 ALK+ ALCL patients receiving chidamide were included in this study and ALK+ ALCL alone has a better prognosis than other subtypes. Given that HDAC inhibitors can impair DNA repair mechanisms, thereby inducing DNA damage, the effects of HDAC inhibitors may be synergistic with the effects of chemotherapy. Several studies have shown that HDAC inhibitors combined with chemotherapy constitute an efficient treatment for PTCL patients, yet the optimal combination regimen remains unknown. This study found that the ORR and DCR were 51.18 and 74.02%, respectively, for patients receiving chidamide combined with chemotherapy (*n* = 127). For patients with an International Prognostic Index (IPI) of 2–3, the ORR in the chidamide combined with chemotherapy group (*n* = 55) was 58% higher than that in the chidamide single-agent group (*n* = 141), with an ORR of 41% (*P* = 0.0031). Chidamide combined with chemotherapy also increased the ORR for patients with an IPI of 4–5 (*n* = 26) relative to the ORR of patients receiving chidamide alone (*n* = 40) with ORRs of 42 and 10%, respectively (*P* = 0.006). The results of a subgroup analysis showed that the ORRs for patients receiving chidamide combined with cyclophosphamide, doxorubicin, vincristine, and prednisone (CHOP)-like regimens, platinum-containing regimens, and other regimens were 53.13, 45.83, and 55.32%, respectively, with DCRs of 81.25, 66.67, and 76.60%, respectively (Table 1).

**Table 1:** Tumor response of different pathologic subtypes

	AITL	ALK unknown ALCL	ALK+ ALCL	ALK− ALCL	ENKL	PTCL others	PTCL-NOS	All
Chidamide alone								
ORR *n* (%)	32 (49.23)	4 (44.44)	4 (66.67)	3 (37.50)	5 (15.15)	5 (55.56)	47 (37.30)	100 (39.06)
CR *n* (%)	6 (9.23)	1 (11.11)	4 (66.67)	2 (25.00)	2 (6.06)	1 (11.11)	11 (8.73)	27 (10.55)
PR *n* (%)	26 (40.00)	3 (33.33)	0 (0.00)	1 (12.50)	3 (9.09)	4 (44.44)	36 (28.57)	73 (28.52)
DCR *n* (%)	49 (75.38)	6 (66.67)	5 (83.33)	6 (75.00)	14 (42.42)	6 (66.67)	79 (62.70)	165 (64.45)
Chidamide combined with chemotherapy regimens								
ORR *n* (%)	25 (71.43)	1 (33.33)	2 (100.00)	1 (14.29)	8 (40.00)	3 (75.00)	25 (44.64)	65 (51. 18)
CR *n* (%)	4 (11.43)	0 (0.00)	1 (50.00)	0 (0.00)	2 (10.00)	1 (25.00)	7 (12.50)	15 (11.81)
PR *n* (%)	21 (60.00)	1 (33.33)	1 (50.00)	1 (14.29)	6 (30.00)	2 (50.00)	18 (32.14)	50 (39.37)
DCR *n* (%)	31 (88.57)	1 (33.33)	2 (100.00)	5 (71.43)	10 (50.00)	4 (100.00)	41 (73.21)	94 (74.02)
Combined with CHOP-like regimens								
ORR *n* (%)	7 (77.78)	1 (50.00)	0 (0.00)	0 (0.00)	1 (33.33)	2 (100.00)	6 (40.00)	17 (53.13)
CR *n* (%)	2 (22.22)	0 (0.00)	0 (0.00)	0 (0.00)	1 (33.33)	1 (50.00)	0 (0.00)	4 (12.50)
PR *n* (%)	5 (55.56)	1 (50.00)	0 (0.00)	0 (0.00)	0 (0.00)	1 (50.00)	6 (40.00)	13 (40.63)
DCR *n* (%)	9 (100.00)	1 (50.00)	0 (0.00)	1 (100.00)	2 (66.67)	2 (100.00)	11 (73.33)	26 (81.25)
Combined with platinum-containing regimens								
ORR *n* (%)	9 (75.00)	0 (0.00)	1 (100.00)	0 (0.00)	3 (42.86)	0 (0.00)	9 (37.50)	22 (45.83)
CR *n* (%)	0 (0.00)	0 (0.00)	0 (0.00)	0 (0.00)	0 (0.00)	0 (0.00)	4 (16.67)	4 (8.33)
PR *n* (%)	9 (75.00)	0 (0.00)	1 (100.00)	0 (0.00)	3 (42.86)	0 (0.00)	5 (20.83)	18 (37.50)
DCR *n* (%)	11 (91.67)	0 (0.00)	1 (100.00)	2 (66.67)	3 (42.86)	0 (0.00)	15 (62.50)	32 (66.67)
Combined with other regimens								
ORR *n* (%)	9 (64.29)	0 (0.00)	1 (100.00)	1 (33.33)	4 (40.00)	1 (50.00)	10 (58.82)	26 (55.32)
CR *n* (%)	2 (14.29)	0 (0.00)	1 (100.00)	0 (0.00)	1 (10.00)	0 (0.00)	3 (17.65)	7 (14.89)
PR *n* (%)	7 (50.00)	0 (0.00)	0 (0.00)	1 (33.33)	3 (30.00)	1 (50.00)	7 (41.18)	19 (40.43)
DCR *n* (%)	11 (78.57)	0 (0.00)	1 (100.00)	2 (66.67)	5 (50.00)	2 (100.00)	15 (88.24)	36 (76.60)

*PTCL-NOS* peripheral T cell lymphoma-not otherwise specified, *AITL* angioimmunoblastic T cell lymphoma, *ENKL* extranodal natural killer/T cell lymphoma, *ALCL* anaplastic large cell lymphoma, *ORR* overall response rate, *DCR* disease control rate

**Fig. 1:**
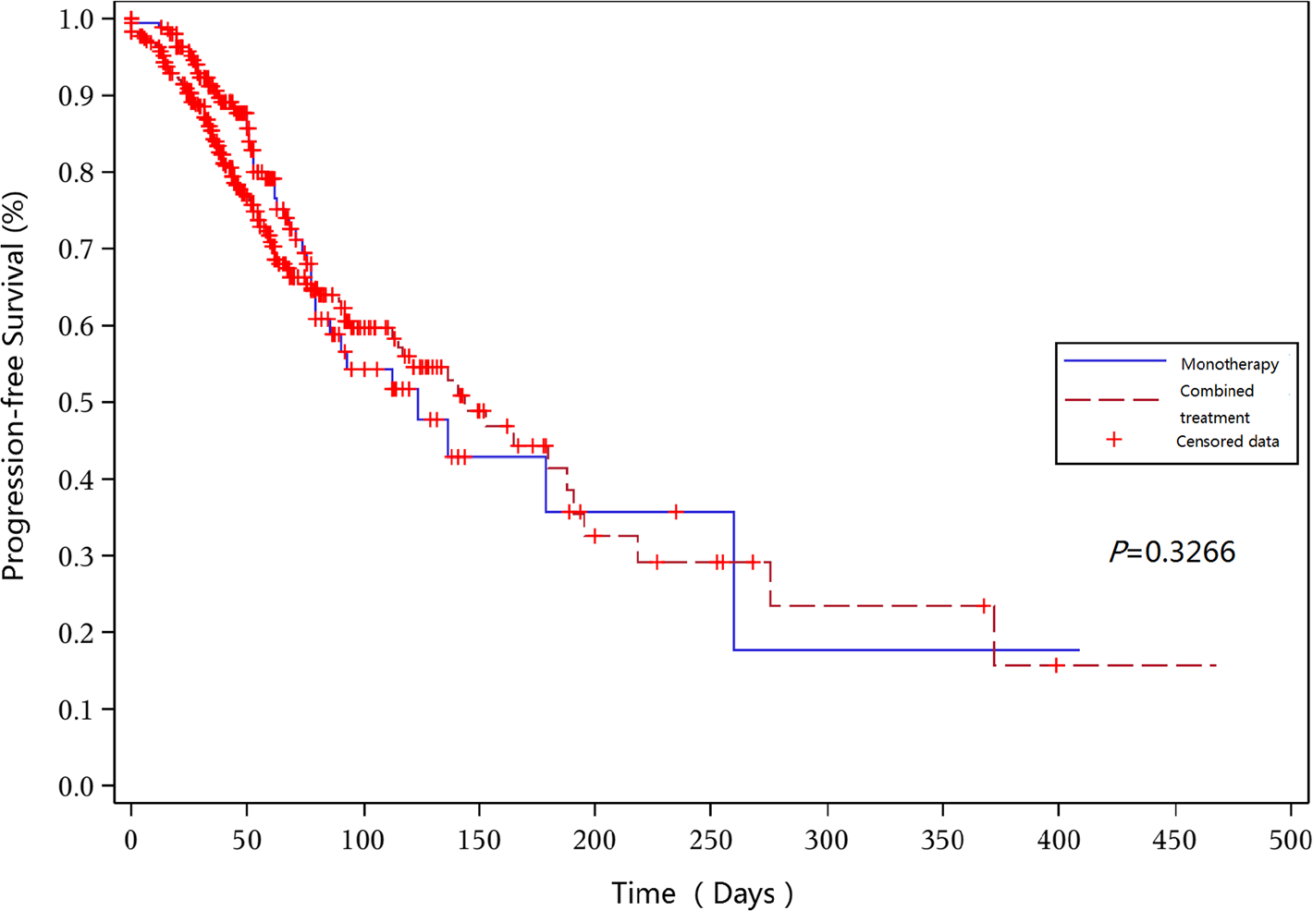
Progression-free survival for patients receiving chidamide monotherapy and patients receiving chidamide combined with chemotherapy

For patients receiving chidamide monotherapy and chidamide combined with chemotherapy, the median progression-free survival (PFS) was 129 (95% CI 82 to 194) days and 152 (95% CI 93 to 201) days, respectively (*P* = 0.3266) (Fig. 1) and the median duration of response (DOR) was 148 (95% CI 132 to 171) days and 169 (95% CI 154 to 192) days, respectively (*P* = 0.3215). In the chidamide monotherapy group, the PFS for AITL and peripheral T cell lymphoma-not otherwise specified (PTCL-NOS) patients were 144.5 days and 133 days, respectively. In the combination group, the PFS for AITL and PTCL-NOS patients were 176 days and 124 days, respectively. The results of a subgroup analysis showed that the median PFS for patients receiving chidamide combined with CHOP-like regimens, platinum-containing regimens, and other regimens was 172, 119, and 160 days, respectively. The median DOR for patients receiving chidamide combined with CHOP-like regimens, platinum-containing regimens, and other regimens was 180, 165, and 172 days, respectively.

Drug-related adverse events (AEs) that occurred in ≥5% of patients receiving chidamide alone included thrombocytopenia (25.0%), neutropenia (19.1%), fatigue (18.4%), nausea/vomiting (14.1%), and anemia (11.3%). Drug-related AEs that occurred in ≥5% of patients receiving chidamide combined with chemotherapy included thrombocytopenia (28.4%), neutropenia (25.2%), fatigue (24.4%), anemia (17.3%), nausea/vomiting (12.7%), increased alanine aminotransferase (ALT) (9.5%), and increased aspartate aminotransferase (AST) (6.3%). Most AEs were of grade 1 to 2. AEs of grade 3 or higher that occurred in ≥5% of patients receiving chidamide alone included thrombocytopenia (10.2%) and neutropenia (6.2%). For patients receiving chidamide combined with chemotherapy, grade 3 to 4 AEs that occurred in ≥5% of patients included thrombocytopenia (18.1%), neutropenia (12.6%), anemia (7.1%), and fatigue (5.5%) (Additional file 3).

In summary, this real-world study conducted with 383 patients demonstrates that chidamide has a favorable efficacy and an acceptable safety profile for refractory and relapsed PTCL patients, confirming the pivotal phase II study in a more representative real-world population. Moreover, this study indicated the potential benefit of chidamide when combined with chemotherapy, which had not been previously examined. Chidamide combined with chemotherapy may be a new treatment choice for PTCL, especially for PTCL patients with an IPI ≥2, although further investigation is warranted.

## Additional files

Additional file 1: Methods (DOCX 16 kb)
Additional file 2: Table S1. Patients’ baseline characteristics (DOCX 16 kb)
Additional file 3: Table S2. Drug-related adverse events in ≥5% of patients (DOCX 21 kb)

## Abbreviations

AEs: Adverse events
ALT: Alanine aminotransferase
ASCT: Autologous stem cell transplantation
AST: Aspartate aminotransferase
CFDA: China Food and Drug Administration
DCR: Disease control rate
DOR: Duration of response
FDA: Food and Drug Administration
HDAC: Histone deacetylase
IPI: International Prognostic Index
NHL: Non-Hodgkin’s lymphoma
ORR: Overall response rate
PFS: Progression-free survival
PTCL: Peripheral T cell lymphoma
