# Supplementary material for: Chidamide in relapsed or refractory peripheral T cell lymphoma: a multicenter real-world study in China
Source: J Hematol Oncol. 2017 Mar 15;10:69. doi: 10.1186/s13045-017-0439-6 (PMC5351273; doi:10.1186/s13045-017-0439-6)
Supplement: Additional file 1: — Methods (DOCX 16 kb) [file 13045_2017_439_MOESM1_ESM.docx]

**Additional File 1**

**Methods**

**Patients and study design**

This was a multicenter real-world study of chidamide in relapsed or refractory peripheral T cell lymphoma (PTCL) in mainland China. Chidamide (5 mg) tablets were provided by Chipscreen Biosciences Ltd. The eligibility criteria were as follows: (1) patients who were diagnosed with AITL, ALCL, extranodal natural killer/T cell lymphoma (ENKL), PTCL-NOS, and other subtypes of PTCL by histopathology; (2) relapsed or refractory status after previous systemic therapy; (3) prescribed chidamide monotherapy or chidamide combined with chemotherapy regimens; and (4) agreed to receive follow up by researchers. Decisions about the treatment modality were made by physicians in each center. A dose of 30 mg chidamide was orally administered twice weekly until disease progression or unacceptable toxicity. The primary endpoint of efficacy evaluation was the overall response rate (ORR) and disease control rate (DCR). The secondary endpoint of efficacy evaluation included the duration of response (DOR) and progression-free survival (PFS). Safety assessments mainly included hematological toxicity, general status, gastrointestinal reaction, liver function injury and cardiotoxicity. Efficacy and safety information were collected at each visit.

**Assessments**

Efficacy was assessed by treatment response and PFS. The treatment response was evaluated according to International Workshop Criteria. The ORR was defined as the complete response/complete response undefined (CR/CRu) rate plus the partial response (PR) rate. The DCR was defined as the CR rate plus PR rate plus stable disease (SD) rate. The PFS was considered to be the time from the date of treatment initiation to the date of disease progression, death or last follow-up. The DOR was defined as the time from the date of a response to the date of disease progression or last follow-up. Disease response was assessed every two treatment cycles. Safety data were assessed based on drug-related adverse events (AEs). AEs were graded by the National Cancer Institute (NCI) Common Terminology Criteria for Adverse Events version 4.03 (CTCAEv4.03).

**Statistical analysis**

Statistical analyses were performed using the SAS 9.0 software package. PFS and DOR were analyzed using the Kaplan-Meier method. A comparison of ORRs was performed using a χ2 test.
