# Supplementary material for: Chidamide in relapsed or refractory peripheral T cell lymphoma: a multicenter real-world study in China
Source: J Hematol Oncol. 2017 Mar 15;10:69. doi: 10.1186/s13045-017-0439-6 (PMC5351273; doi:10.1186/s13045-017-0439-6)
Supplement: Additional file 2: Table S1. — Patients’ baseline characteristics (DOCX 16 kb) [file 13045_2017_439_MOESM2_ESM.docx]

| **Table S1. Patients' baseline characteristics** | | |
| --- | --- | --- |
| Characteristics | N=383 | |
|  | N | (%) |
| Age, years |  | |
| Median | 55 | |
| Range | 18-88 | |
| Gender^*^ |  |  |
| Male | 254(379) | 67.02% |
| Female | 125(379) | 32.98% |
| ECOG^*^ |  |  |
| 0 | 75(314) | 23.89% |
| 1 | 107(314) | 34.08% |
| ≥2 | 132(314) | 42.03% |
| Stage^*^ |  |  |
| I | 9(366) | 2.46% |
| II | 22(366) | 6.01% |
| III | 125(366) | 34.15% |
| IV | 210(366) | 57.38% |
| Pathology |  |  |
| PTCL-NOS | 182 | 47.52% |
| AITL | 100 | 26.11% |
| ENKL | 53 | 13.84% |
| ALCL | 35 | 9.14% |
| PTCL other types | 13 | 3.39% |
| IPI^*^ |  |  |
| 0 | 12(325) | 3.69% |
| 1 | 51(325) | 15.69% |
| 2 | 89(325) | 27.38% |
| ≥3 | 173(325) | 53.24% |
| Treatment |  |  |
| Chidamide alone | 256 | 66.84% |
| Combined with  chemotherapy | 127 | 33.16% |
| CHOP-like | 32 | 8.36% |
| Platinum  containing | 48 | 12.53% |
| Other regimens | 47 | 12.27% |
| ^*^Gender could be evaluated in 379 patients; ECOG could be evaluated in 314 patients; Stage could be evaluated in 366 patients; IPI could be evaluated in 325 patients.  ECOG, Eastern Cooperative Oncology Group; PTCL-NOS, peripheral T cell lymphoma-not otherwise specified; AITL, angioimmunoblastic T cell lymphoma; ENKL, extranodal natural killer/T cell lymphoma; ALCL, anaplastic large cell lymphoma; IPI, International Prognostic Index | | |
