# Supplementary material for: Chidamide in relapsed or refractory peripheral T cell lymphoma: a multicenter real-world study in China
Source: J Hematol Oncol. 2017 Mar 15;10:69. doi: 10.1186/s13045-017-0439-6 (PMC5351273; doi:10.1186/s13045-017-0439-6)
Supplement: Additional file 3: Table S2. — Drug-related adverse events in ≥5% of patients (DOCX 21 kb) [file 13045_2017_439_MOESM3_ESM.docx]

| **Table S2. Drug-related adverse events in ≥5% of patients** | | | | | | |
| --- | --- | --- | --- | --- | --- | --- |
| Events | Grade 1 | Grade 2 | Grade 3 | Grade 4 | Total | Grades 3-4 |
| Chidamide alone (N=256) | | | | | | |
| Hematological toxicity |  |  |  |  |  |  |
| Anemia | 6(2.3) | 14(5.5) | 6(2.3) | 3(1.2) | 29(11.3) | 9(3.5) |
| Neutropenia | 10(3.9) | 23(9.0) | 9(3.5) | 7(2.7) | 49(19.1) | 16(6.2) |
| Thrombocytopenia | 10(3.9) | 28(10.9) | 16(6.3) | 10(3.9) | 64(25.0) | 26(10.2) |
| Fatigue | 16(6.3) | 19(7.4) | 12(4.7) | 0(0.0) | 47(18.4) | 12(4.7) |
| Nausea/vomiting | 24(9.4) | 8(3.1) | 4(1.6) | 0(0.0) | 36(14.1) | 4(1.6) |
| Combined with chemotherapy (N=127) | | | | | | |
| Hematological toxicity |  |  |  |  |  |  |
| Anemia | 4(3.1) | 9(7.1) | 7(5.5) | 2(1.6) | 22(17.3) | 9(7.1) |
| Neutropenia | 5(3.9) | 11(8.7) | 9(7.1) | 7(5.5) | 32(25.2) | 16(12.6) |
| Thrombocytopenia | 3(2.4) | 10(7.9) | 9(7.1) | 14(11.0) | 36(28.4) | 23(18.1) |
| Fatigue | 15(11.8) | 9(7.1) | 7(5.5) | 0(0.0) | 31(24.4) | 7(5.5) |
| Nausea/vomiting | 11(8.7) | 2(1.6) | 3(2.4) | 0(0.0) | 16(12.7) | 3(2.4) |
| Liver function injury |  |  |  |  |  |  |
| Increase of ALT | 9(7.1) | 1(0.8) | 1(0.8) | 1(0.8) | 12(9.5) | 2(1.6) |
| Increase of AST | 7(5.5) | 0(0.0) | 1(0.8) | 0(0.0) | 8(6.3) | 1(0.8) |
| ALT, alanine aminotransferase; AST, aspartate aminotransferase | | | | | | |

| **Table S3. Comparison of drug-related adverse events in ≥5% of patients in different treatment groups** | | | | | | | | |
| --- | --- | --- | --- | --- | --- | --- | --- | --- |
|  | Total | | | | Grades 3-4 | | | |
|  | CHOP-like regimens (N=32) | Platinum-containing regimens (N=48) | Other regimens  (N=47) | P | CHOP-like regimens (N=32) | Platinum-containing regimens (N=48) | Other regimens  (N=47) | P |
| Hematological toxicity |  |  |  |  |  |  |  |  |
| Anemia | 8(25.0%) | 7(14.6%) | 10(21.3%) | 0.4874 | 3(9.4%) | 2(4,2%) | 5(10.6%) | 0.4564* |
| Neutropenia | 10(31.3%) | 13(27.1%) | 12(25.5%) | 0.8519 | 6(18.8%) | 6(12.5%) | 5(10.6%) | 0.5676 |
| Thrombocytopenia | 13(40.6%) | 13(27.1%) | 14(29.8%) | 0.4205 | 9(28.1%) | 8(16,7%) | 7(15.0%) | 0.2974 |
| Fatigue | 10(31.3%) | 11(23.0%) | 12(25.5%) | 0.7043 | 4(12.5%) | 3(6.3%) | 0(0.0%) | 0.0465* |
| Nausea/vomiting | 3(9.4%) | 6(12.5%) | 8(17.0%) | 0.6028 | 0(0.0%) | 1(2.1%) | 2(4.3%) | 0.6244* |
| Liver function injury |  |  |  |  |  |  |  |  |
| Increase of ALT | 4(12.5%) | 4(8.3%) | 6(12.8%) | 0.7517 | 0(0.0%) | 0(0.0%) | 1(2.1%) | 0.6220* |
| Increase of AST | 2(6.3%) | 2(4.2%) | 5(10.6%) | 0.4671* | 0(0.0%) | 0(0.0%) | 0(0.0%) | - |
| ALT, alanine aminotransferase; AST, aspartate aminotransferase  *Fisher's exact test | | | | | | | | |
